# Factors associated with parents' willingness to vaccinate their children against COVID-19: The LA pandemic surveillance cohort study

**DOI:** 10.3934/publichealth.2022033

**Published:** 2022-05-25

**Authors:** Chun Nok Lam, William Nicholas, Alejandro De La Torre, Yanpui Chan, Jennifer B. Unger, Neeraj Sood, Howard Hu

**Affiliations:** 1 Keck School of Medicine, University of Southern California, Los Angeles, USA; 2 Los Angeles County Department of Public Health, Los Angeles, USA; 3 Sol Price School of Public Policy, University of Southern California, Los Angeles, USA

**Keywords:** COVID-19, vaccination, hesitancy, parent, children

## Abstract

**Background:**

Children age 5–11 became eligible for COVID-19 vaccination in November 2021 in the United States, but vaccine uptake in this age group remains low. Understanding reasons why parents are hesitant to vaccinate their children may provide critical insights to help protect children from COVID-19 infection. This study examines factors associated with parents' willingness to vaccinate their children.

**Methods:**

We conducted a cross-sectional survey focusing on the Los Angeles County adult residents between March and June 2021. Our analytic sample focused on a subgroup of participants who self-report having a child. Predictors included parents' vaccination status and beliefs about COVID-19. We used multivariable logistic regression analysis and calculated the predicted probabilities of parents' willingness to vaccinate their children.

**Results:**

Parents (n = 401) who worried about catching the virus, had trust in vaccine development and the COVID-19 vaccine approval process, and vaccinated against COVID-19 were more likely to be willing to vaccinate their children. Socio-economic, racial and ethnic differences were no longer statistically significant in the adjusted model. Predicted probabilities of parents who were willing to vaccine their children were 55% among the vaccinated and 36% among the unvaccinated.

**Conclusions:**

Parents' intent to vaccinate their children is influenced by their perceived severity of the pandemic, trust in the vaccine development process, and their vaccination status, which can be the potential drivers of hesitancy to vaccinate their children.

## 1. Introduction

Children age 5–11 became eligible for COVID-19 vaccination in the US in November 2021, but vaccine uptake in this age group remains low [1]. A recent poll from the Kaiser Family found that only 34% of parents or caregivers said they would vaccinate their 5 to 11-year-old children immediately after vaccine authorization for this age group [2]. Children typically present with less severe clinical manifestations of SARS-CoV-2, while some can become very ill [3]. In addition, children can spread COVID-19 to others [4]. Understanding reasons why parents or caregivers are hesitant to vaccinate their children may provide critical insights to help protect children from COVID-19 infection.

Among studies of U.S. parents' intent to vaccinate their children against COVID-19 [5]–[9], 34–63% reported they were likely to vaccinate their children. Three U.S. national surveys found that parents who were younger, less educated, racial/ethnic minorities, had lower income, had younger children, and were not vaccinated against COVID-19 themselves were less likely to plan to vaccinate their children against COVID-19 [5],[6],[9],[10]. Lendon et al. found that mistrust in government and vaccines was the most common reason for parents who definitely would not get their children vaccinated against COVID-19 [10]. Szilagyi et al. found similar associations and found that the child's doctor was a key trusted source of COVID-19 information [6]. Rhodes et al. found that vaccine hesitant parents conducted their own research on vaccination using sources they trust [5]. As disparities in adult COVID-19 vaccination exist, it is plausible to expect similar disparities in parents' intentions to vaccinate their children. And if in populations that are already experiencing health disparities refuse vaccination, the health disparities could widen.

This study examines factors associated with parents' intent to vaccinate their children in Los Angeles County, California. We hypothesize that parents' demographic characteristics, trust in vaccine development and perceived susceptibility to COVID-19 will be significant predictors. We calculated predicted probabilities of parents' willingness to vaccinate children by their own vaccination status and beliefs about COVID-19. Our results help elucidate the differential effects of factors that influence parents' willingness to vaccinate children, which can lead to improved targeted health communication messages to promote child vaccination.

## 2. Materials and methods

### 2.1. Study sample

An online survey was conducted as part of a larger study focusing on the seroprevalence of COVID-19 antibodies in a representative sample of Los Angeles County population [11]. LRW, A Material Company, implemented the recruitment process and sent the questionnaire to its online member platform. The survey was conducted between March and June 2021, when the vaccine was primarily available for age 65+ and among essential workers and early on for age 16+. The questionnaire was available in English and Spanish. All participants provided written informed consent. The analytic sample focused on a subgroup of participants who self-report having a child, as defined as the parents in this study. The Los Angeles County Department of Public Health Institutional Review Board approved all study procedures. Survey items used in the study analysis can be accessed in the Supplementary Material S1.

### 2.2. Measure

**Figure 1.**
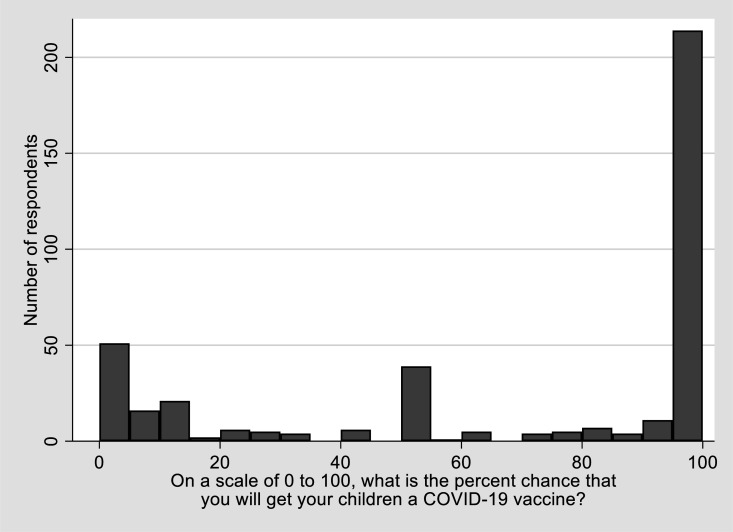
Distribution of study outcome. The figure shows that half of the respondents (n = 204) indicated >95 percent chance that they will get their children a COVID-19 vaccine. The analysis used n = 195 who indicated 100 percent chance as the dichotomous cut-off for willingness to vaccinate children based on the outcome distribution as shown.

The primary outcome asked “On a scale of 0 to 100, what is the percent chance that you will get your children a COVID-19 vaccine?”. Because nearly half of the respondents reported 100 on the scale, we dichotomized the response to 100 vs <100 (1 vs 0) as likely vs less than likely to be willing to vaccine their children (Figure 1). We conducted sensitivity analyses with cutoffs at 90 and 80 to ensure the result was not affected. Study covariates included self-report age, sex, race/ethnicity, household income, healthcare access, COVID-19 testing and vaccination status. Other predictors include perceived susceptibility to COVID-19 infection, access to COVID-19 information, and trust in the vaccine development and approval process (Yes vs No for each item).

### 2.3. Statistical analysis

We conducted descriptive, univariate and multivariable logistic regression analyses to test factors associated with parents' willingness to vaccinate their children. We used the multivariable regression models to estimate adjusted odds ratios and marginal effects (change in predicted probability of vaccination) for each key covariate [12]. We performed all statistical tests in Stata 15 with α set to 0.05.

## 3. Results

Of the 1222 adults who completed the questionnaire, 401 indicated being a parent and answered the study outcome. Overall, 48.6% of parents were willing to vaccinate their children. Participants were more female (63.3%), middle to older age range (35–49: 37.2%, ≥50: 43.9%), White or Hispanic individuals (34.2%, 36.7%), had higher income (≥$100,000: 42.1%), ever tested for COVID-19 (81.8%), vaccinated against COVID-19 (65.8%), and had regular healthcare access (84.5%) (Table 1).

In the univariate analyses, being a non-Hispanic White parent (54.7% vs Hispanic: 36.1% and non-Hispanic Black: 42.4%, p = 0.04 and 0.05), having higher income (≥$100k: 58.0% vs <$50k: 36.5%, p < 0.01), regular healthcare access (51.0% vs 35.5%, p = 0.03), and being vaccinated against COVID-19 (59.1% vs 28.5%, p < 0.01) were more likely to be willing to vaccinate their children (Table 1).

**Table 1.** Parents' willingness to vaccinate their children.

Characteristics	Total sample, n	Willing to Vaccinate Children, n (%)	Unadjusted OR (95% CI)	p-value	Adjusted OR (95% CI)	p-value
Overall Sample	401	195 (46.8%)				
Sex
Female	254	120 (47.2%)	Ref		Ref	
Male	147	75 (51.0%)	1.2 (0.8, 1.7)	0.47	0.7 (0.4, 1.2)	0.23
Age, y
18–34	76	33 (43.4%)	Ref		Ref	
35–49	149	63 (42.3%)	1.0 (0.5, 1.7)	0.87	0.7 (0.4, 1.5)	0.38
≥50	176	99 (56.3%)	1.7 (1.0, 2.9)	0.06	1.2 (0.6, 2.4)	0.63
Race/ethnicity
White (non-Hispanic)	137	75 (54.7%)	Ref		Ref	
Hispanic	147	62 (42.2%)	0.6 (0.4, 1.0)	0.04	0.8 (0.5, 1.5)	0.57
Black (non-Hispanic)	36	13 (36.1%)	0.5 (0.2, 1.0)	0.05	0.8 (0.4, 2.0)	0.68
Asian (non-Hispanic)	59	33 (55.9%)	1.0 (0.6, 1.9)	0.88	1.5 (0.7, 3.2)	0.26
Other	22	12 (54.6%)	1.0 (0.4, 2.4)	0.27	0.7 (0.2, 2.2)	0.54
Income, Annual Household
<$50,000	96	35 (36.5%)	Ref		Ref	
$50,000–$99,999	120	58 (48.3%)	1.6 (0.9, 2.8)	0.08	1.1 (0.6, 2.1)	0.81
≥100,000	169	98 (58.0%)	2.4 (1.4, 2.0)	<0.01	1.3 (0.7, 2.6)	0.40
Prefer not to answer	16	4 (25.0%)	0.6 (0.2, 1.9)	0.38	0.5 (0.1, 1.8)	0.26
Regular healthcare access
No	62	22 (35.5%)	Ref		Ref	
Yes	339	173 (51.0%)	1.9 (1.1, 3.3)	0.03	1.2 (0.6, 2.4)	0.63
Tested for COVID-19, ever
No	73	33 (45.2%)	Ref		Ref	
Yes	328	162 (49.4%)	1.2 (0.7, 2.0)	0.52	1.1 (0.6, 2.0)	0.77
Vaccinated for COVID-19, ever
No	137	39 (28.5%)	Ref		Ref	
Yes	264	156 (59.1%)	3.6 (2.3, 5.7)	<0.01	2.9 (1.7, 4.9)	<0.01
Worry about catching the virus^a^
No	265	131 (49.4%)	Ref		Ref	
Yes	136	64 (47.1%)	0.9 (0.6, 1.4)	0.65	2.0 (1.1, 3.8)	0.03
Worry that I can't keep my family safe from the virus^a^		
No	261	135 (51.7%)	Ref		Ref	
Yes	140	60 (42.9%)	0.7 (0.7, 1.1)	0.09	0.8 (0.4, 1.5)	0.54
Searched the Internet for treatments for COVID-19^b^		
No	153	153 (53.5%)	Ref		Ref	
Yes	115	42 (36.5%)	0.5 (0.3, 0.8)	<0.01	0.6 (0.3, 1.1)	0.11
Asked health professionals for advice about COVID-19^b^		
No	138	142 (54.0%)	Ref		Ref	
Yes	263	53 (38.4%)	0.5 (0.3, 0.8)	<0.01	0.6 (0.3, 1.2)	0.12
Trust vaccine development process in general^c^		
No	262	85 (32.4%)	Ref		Ref	
Yes	139	110 (79.1%)	7.9 (4.9, 12.8)	<0.01	3.8 (1.7, 8.2)	<0.01
Trust government approval process for COVID-19 vaccine^c^		
No	272	93 (34.2%)	Ref		Ref	
Yes	129	102 (79.1%)	7.3 (4.4, 11.9)	<0.01	2.6 (1.2, 5.7)	0.02

*Note: ^a^Yes: extremely worried, very worried, moderately worried; No: slightly worried, not at all worried. ^b^Yes: almost always, often, sometimes, rarely; No: never. ^c^Yes: fully trust; No: mostly trust, somewhat trust, do not trust.

In the multivariable model, demographic characteristics were not statistically significant. Parents' COVID-19 vaccination status (AOR: 2.9, 95% CI: 1.7, 4.9, p < 0.01), being worried about catching the virus (AOR: 2.0, 95% CI: 1.1, 3.8, p = 0.03), trusting vaccine development process in general (AOR: 3.8, 95% CI: 1.7, 8.2, p < 0.01), and trusting the government approval process for COVID-19 vaccine (AOR: 2.6, 95% CI: 1.2, 5.7, p = 0.02) were significant predictors for being willing to vaccinate their children.

**Figure 2.**
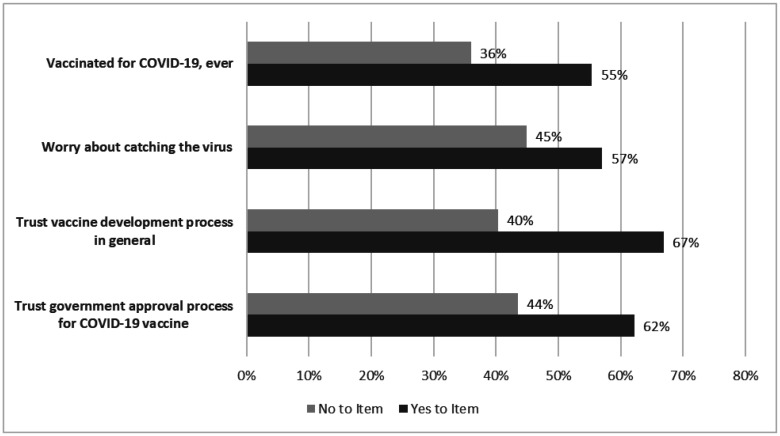
Predicted probabilities of parents who were willing to vaccinate their children. The horizontal bars show the predicted probabilities of parents who were willing to vaccinate their children, based on parents' response to the four study items. For example, among parents who were ever vaccinated for COVID-19, 55% were predicted to be willing to vaccinate their children, as oppose to among parents who were not vaccinated for COVID-19, 36% were predicted to be willing to vaccinate their children.

The predicted probabilities of parents who were willing to vaccinate their children were 55.4% (95 CI: 49.9, 60.9) among vaccinated parents and 36.0% (95% CI: 28.6, 43.4) among the unvaccinated parents; 57.0% (95% CI: 48.6, 65.4) among those who worry about catching the virus and 44.9% (95% CI: 39.7, 50.0) among those who do not worry; 66.9% (95% CI: 55.4, 78.3) among those who trust vaccine development in general and 40.3% (95% CI: 33.2, 47.5) among those who do not trust; and 62.2% (95% CI: 49.4, 74.2) among those who trust the government approval process for COVID-19 vaccine and 43.5% (95% CI: 37.0, 50.0) among those who do not trust (Figure 2).

## 4. Discussion

Our study adds several insights to the existing literature. First, among vaccinated parents, only 55% were willing to vaccinate their children; among unvaccinated the probability drops to 36%. Second, socio-demographic characteristics were not significant predictors after adjusting for parents' beliefs on COVID-19. This suggests that parents' beliefs, including their trust of the vaccine development and approval process may be the primary drivers of vaccine hesitancy for their children instead of parents' own race and ethnicity. Parents' attitudes about vaccination, including their own vaccination status, can be consistent across members of their family, and can also affect their children's access to the COVID-19 vaccine.

Our research suggests that educational interventions that focus on improving trust in vaccine development processes in general and for COVID-19 vaccines in particular will likely boost vaccination rates among children. The research also suggests that new information on effects of COVID-19 on children will also likely effect vaccination rates. It is important to provide accurate information on COVID-19 risks to parents so that they can make informed decisions. Another approach is the review of American Association Pediatrics and CDC guidelines related to the efficacy and safety of COVID-19 vaccine and vaccines in general to address parental concerns during their child's clinical encounter [13]. Finally, vaccine mandates for children such as the proposed COVID-19 vaccine mandate for all California school age children may have large unintended consequences. We find that among vaccinated parents, about 45% are hesitant to vaccinate their children and two-thirds of unvaccinated parents are hesitant to vaccinate their children. Thus, it is possible that even with a mandate significant fraction of parents might choose to have their children remain unvaccinated. These unvaccinated children would not be allowed to attend school, adversely affecting their later life outcomes.

Our findings reflect on one of the US's most racial and ethnically diverse metropolitan areas, the Los Angeles County, about parents' views on child vaccination against COVID-19, in the months immediately before the vaccine was authorized for children aged 12–15 [14]. Given that COVID-19 vaccine is now available for children aged 5–17, studies can examine disparities in actual vaccination uptake in this age groups, and factors that motivated once vaccine hesitant parents who then completed the COVID-19 vaccination for their children.

## 5. Limitations

This study has several limitations. First, we did not collect demographic data from the children of surveyed parents, including age and sex of the children. Second, only a subgroup of the representative sample from the larger study were self-report parents, which can limit the generalizability of our findings. Third, results from the sensitivity analysis using more inclusive cutoffs for the outcome variable showed that a key predictor, worry about catching the virus, did not remain statistically significant at these lower cutoffs. This suggests that worry about catching the virus only predicted parents' willingness to vaccinate their children at the highest vs less than highest level of intent (100 vs <100). Nevertheless, other predictors remained unaffected by the cutoffs.

## 6. Conclusions

Parents' willingness to vaccinate their children is influenced by their vaccination status, perceived severity of the pandemic and trust in the COVID-19 vaccine approval process. Public health strategies will need to focus on a timely and effective solution to address the disparities in vaccination uptake among young children.
