# Supplementary material for: Factors associated with parents' willingness to vaccinate their children against COVID-19: The LA pandemic surveillance cohort study
Source: AIMS Public Health. 2022 May 25;9(3):482–9. doi: 10.3934/publichealth.2022033 (PMC9581747; doi:10.3934/publichealth.2022033)
Supplement: Supplementary file 1 [file publichealth-09-03-033-s001.pdf]

AIMS Public Health, 9(3): 482–489.
DOI: 10.3934/publichealth.2022033
Received: 21 March 2022
Revised: 28 April 2022
Accepted: 15 May 2022
Published: 25 May 2022

http://www.aimspress.com/journal/aimsph

Research article

Factors associated with parents' willingness to vaccinate their children against COVID-19: The LA pandemic surveillance cohort study

Chun Nok Lam[1,*], William Nicholas[2], Alejandro De La Torre[1], Yanpui Chan[1], Jennifer B. Unger[1], Neeraj Sood[3] and Howard Hu[1]

[1] Keck School of Medicine, University of Southern California, Los Angeles, USA
[2] Los Angeles County Department of Public Health, Los Angeles, USA
[3] Sol Price School of Public Policy, University of Southern California, Los Angeles, USA

* **Correspondence:** Email: chunnok.lam@med.usc.edu.

Survey questions used in the manuscript

Please indicate your gender
Male
Female
Transgender
I prefer a different term (please specify)
Please enter your date of birth
ENTER DATE: MM/DD/YYYY
Prefer not to answer
Are you Hispanic, Latino/a, or of Spanish origin? ***(Please select all that apply)***
No, not of Hispanic, Latino or Spanish origin
Yes, Mexican, Mexican American, Chicano
Yes, Central American (Please specify)
Yes, South American (Please specify)
Yes, another Hispanic, Latino or Spanish origin from Spain
Prefer not to answer
Please indicate your race
White or Caucasian
Black or African American

American Indian or Alaska Native
Asian or Asian American
Native Hawaiian or Pacific Islander
Other (please specify)
Prefer not to answer

What was your household income from all sources before taxes in the past 12 months? Please provide your best estimate.

Under $10,000
$10,000 to $24,999
$25,000 to $49,999
$50,000 to $74,999
$75,000 to $99,999
$100,000 to $149,999
$150,000 to $199,999
$200,000 and over
Prefer not to answer

Do you have one place that you regularly go to when you need health care or have a question about your or your family's health?

Yes
No

Have you <u>ever</u> been tested for COVID-19?

Yes
No
Prefer not to answer

Have you been vaccinated against COVID-19?

Yes
No
Not sure

In the <u>past 7 days</u>, how worried were you…?

…about catching the virus
…that I can't keep my family safe from the virus
Not at all worried
Slightly worried
Moderately worried
Very worried
Extremely worried

The following questions ask about health seeking behaviors. <u>During the PAST 7 DAYS</u>, how often have you done the following because of concerns about COVID-19?

…Searched the Internet for treatments for COVID-19
…Asked health professionals (e.g., doctors or pharmacists) for advice about COVID-19
Never
Rarely
Sometimes
Often

Almost Always

How much do you trust the process in general (not just for COVID-19) to develop safe vaccines for the public?

Fully trust

Mostly trust

Somewhat trust

Do not trust

How much do you trust the governmental approval process to ensure the COVID-19 vaccine is safe for the public?

Fully trust

Mostly trust

Somewhat trust

Do not trust

AIMS Press

© 2022 the Author(s), licensee AIMS Press. This is an open access article distributed under the terms of the Creative Commons Attribution License (http://creativecommons.org/licenses/by/4.0)
